# Impact of weekday and weekend mobility and public policies on COVID-19 incidence and deaths across 76 large municipalities in Colombia: statistical analysis and simulation

**DOI:** 10.1186/s12889-022-14781-7

**Published:** 2022-12-31

**Authors:** Jamie S. Jason, Diana M. Bowser, Arturo Harker Roa, Diana C. Contreras Ceballos, Santiago Muñoz, Anna G. Sombrio, Donald S. Shepard

**Affiliations:** 1 The Heller School for Social Policy and Management, Brandeis University, Waltham, USA; 2 School of Government, Alberto Lleras Camargo, Universidad de Los Andes, Bogotá, Colombia

**Keywords:** COVID-19, Colombia, Mobility, Municipality, Weekend, Population density, Lockdown, Case, Death, SARS-CoV-2

## Abstract

**Background:**

Despite widespread restrictions on residents’ mobility to limit the COVID-19 pandemic, controlled impact evaluations on such restrictions are rare. While Colombia imposed a National Lockdown, exceptions and additions created variations across municipalities and over time.

**Methods:**

We analyzed how weekend and weekday mobility affected COVID-19 cases and deaths. Using GRANDATA from the United Nations Development Program (UNDP) we examined movement in 76 Colombian municipalities, representing 60% of Colombia's population, from March 2, 2020 through October 31, 2020. We combined the mobility data with Colombia’s National Epidemiological Surveillance System (SIVIGILA) and other databases and simulated impacts on COVID-19 burden.

**Results:**

During the study period, Colombians stayed at home more on weekends compared to weekdays. In highly dense municipalities, people moved less than in less dense municipalities. Overall, decreased movement was associated with significant reductions in COVID-19 cases and deaths two weeks later. If mobility had been reduced from the median to the threshold of the best quartile, we estimate that Colombia would have averted 17,145 cases and 1,209 deaths over 34.9 weeks, reductions of 1.63% and 3.91%, respectively. The effects of weekend mobility reductions (with 95% confidence intervals) were 6.40 (1.99–9.97) and 4.94 (1.33–19.72) times those of overall reductions for cases and deaths, respectively.

**Conclusions:**

We believe this is the first evaluation of day-of-the week mobility on COVID-19. Weekend behavior was likely riskier than weekday behavior due to larger gatherings and less social distancing or protective measures. Reducing or shifting such activities outdoors would reduce COVID-19 cases and deaths.

**Supplementary Information:**

The online version contains supplementary material available at 10.1186/s12889-022-14781-7.

## Summary

We analyzed how weekend and weekday mobility affected COVID-19 cases and deaths in Colombia. Using GRANDATA from the UNDP, we examined movement in 76 municipalities, representing 60% of Colombia's population, from March 2, 2020 through October 31, 2020. 

## Introduction

As of October 2022, the COVID-19 pandemic had claimed 6.6 million lives globally based on reports to the World Health Organization (WHO) [1]. Throughout the pandemic, governments, leaders, public health specialists, and medical professionals have promoted numerous public health measures to try to reduce the spread of the SARS‐CoV‐2 virus, the cause of COVID-19. According to the US Centers for Disease Control and Prevention (CDC), “Social distancing helps limit opportunities to come in contact with contaminated surfaces and infected people outside the home. Since people can spread the virus before they know they are sick, it is important to stay at least 6 feet away from others when possible” [2]. As part of this messaging, individuals have been encouraged, and sometimes mandated, to “stay at home” and avoid unnecessary travel to help stop the spread of the virus.

Collecting data and surveying individuals about their self-reported adherence to these restrictions on residents’ mobility and other public health measures has been difficult. Using mobility data collected through mobile phone usage has become a more common way to understand actual mobility patterns throughout the pandemic. This study examines mobility trends across 76 municipalities in Colombia during the COVID-19 pandemic to understand how government “stay-at-home” orders and mobility restrictions impact mobility patterns, new COVID-19 cases, and COVID-19 deaths. In addition, research sponsored by United Nations Development Program (UNDP) noted policy makers’ goal of containing the COVID pandemic while maintaining the formal economy insofar as possible [3].

A number of models have used mobility data publicly collected by groups such as Google, Facebook, and Baidu to predict COVID-19 cases. One study used global mobility trends across countries to estimate the effect of mobility-reducing policies on decreasing new infections over time [4]. Another study used national- and municipal-level mobility data within Mexico to analyze mobility trends to determine how political events influenced mobility patterns [5]. Another study from Japan analyzed how personal knowledge and information regarding COVID-19 impacts decisions to follow or ignore stay-at-home and social distancing measures [6]. None of these studies examined mobility trends across many lower-level government entities within a middle-income country using high frequency data (weekly) over many months.

To our knowledge, this is the first survey collected across multiple municipalities within a country that examines various domains of community trends in mobility during the COVID-19 pandemic. The research was able to combine data on mobility patterns at the municipality level with several other municipal-level databases that capture government social distancing and public health policies, self-reported behaviors, and COVID-19 epidemiological data (cases and deaths) in related studies [7]. The robust database allowed for a detailed examination of mobility patterns; how mobility patterns are impacted by government policies, restrictions, and country characteristics; and how mobility patterns impact COVID-19 cases and deaths. Since the data in this study were collected, COVID-19 vaccines have become widely available. Although Colombia’s share of the population completing the initial (2-dose) vaccination regimen (70.8%) exceeded that for the United States (68.3%), Colombia lagged behind that of its large neighbor, Brazil (80.0%) as of October 14, 2022, the latest common data [8]. Thus, the need for a detailed understanding of the COVID-19 pandemic and a portfolio of control strategies remains.

This study explores several questions using these mobility data from 76 Colombian municipalities over the period March 2020 through October 2020. Altogether, Colombia has 1,122 municipalities, the second level, after departments, in its administrative hierarchy. Our questions include: 1) How do mobility patterns change according to weekday or weekend segments? 2) How do mobility trends differ based on a municipality’s population density? 3) What is the relationship between change in mobility and COVID-19 cases and deaths by municipality?

## Methods

### Mobility data

GRANDATA collected geolocation events of smartphone users using a MADID (Mobile Advertising ID) “hash”. Users with under 10 events a day or recorded within a short time span were dropped from the dataset so that only smartphones with sufficient mobility information were included. For each unique user, the phone's most frequented location was assumed to be the user’s residence, and all other mobility events outside of this residence were labeled as outings.

The mobility indicators captured the amount of human movement that took place in a particular area, measured through an index that compared the level of movement with respect to the benchmark date of March 2, 2020. A value of zero (0) indicated no change in mobility, whereas a value of one (1) indicated a 100% increase in mobility, compared to the benchmark [9]. The mobility index was reported by daily metrics. To standardize comparisons across municipalities in Colombia, data for each municipality were averaged by week to produce a mobility change percentage. These observations date from March 2, 2020 through October 31, 2020, providing 34.97 weeks of data for analysis. To ensure standardized week labeling among various databases, we followed the epidemiological calendar numbering of weeks from Colombia’s National Institute of Health. Week numbering for mobility data in this study started on Week 10 (March 2, 2020) and ended on Week 45. Weeks started on Sunday according to the National Epidemiological Surveillance System (SIVIGILA) calendar [10]. Weekly mobility trends were captured across 76 major municipalities that are home to around 82% of the Venezuelan migrant population and approximately 60% of the country’s entire population [11].

Movement data could potentially measure mobility in intensive (amount of movement per person moving) or extensive terms (share of persons moving). Based on available descriptions of the GRANDATA, these data do not contain separate denominators on mobile phone ownership by municipality. Rather, the data utilized in the analysis below captured movement per phone user. Therefore, the GRANDATA provide the intensive measure, but do not allow calculation of the extensive measure. As the GRANDATA are anonymized and aggregated by municipality by day, they do not describe the movement of any specific population segment. As some households may share a phone among members, the movement in these cases describes the movement of the mobile household member. If, as expected, patterns of sharing phones within a household remain similar or change similarly across all municipalities, the GRANDATA still provide a consistent measure of mobility. If there were major changes in phone sharing over the pandemic, those changes would confound the measure of mobility. Despite this theoretical limitation, mobility by municipality by day provides an important and likely valid measure.

For purposes of the data analysis, weekly mobility trend data were segmented into four categories to account for mobility trends according to different day-of-week segments. The four segments were the average weekly mobility of: 1) Monday and Friday (referred to as shoulder days), 2) Saturday and Sunday (weekend), 3) Tuesday, Wednesday, Thursday (midweek), and 4) weekly (7-day period). Observing mobility patterns by these segments allowed an analysis of how movement throughout the week impacts the spread of COVID-19 and how populations in Colombia responded to COVID-19 safety precautions and restrictions.

### Policy response database

Our analysis examined how mobility trends responded to numerous Colombian subnational COVID-19 public health and social distancing policies. A panel database, referred to as the Policy Response Database [12], was created that extracted dates of implementation for 34 varying policies that were implemented at the sub-national level in Colombia on the following three categories: mobility restriction policies (e.g. capacity restrictions on commercial venues), self-care policies (e.g., mask mandates), and economic support policies (e.g. cash transfers and discounts on public services). Of these three categories, additional information was provided for each measure to further specify regulations of policies. For example, lockdown containment measures have accompanying data specifying the number of hours lockdowns were in place. The database uploaded to GITHUB (San Francisco, CA USA) provides additional details on all the measures.

### Sistema Nacional de Vigilancia en Salud Pública (SIVIGILA) population database

We integrated Colombia’s National Epidemiological Surveillance System, (SIVIGILA) into the master database to gather information on characteristics of those living in Colombia at a municipal level [10]. Population figures were used to calculate new weekly COVID-19 case and death rates per 100,000 inhabitants. The new cases variable was presented as the weekly new cases of COVID-19 divided by the number of inhabitants in a municipality per 100,000 people. The new deaths variable was presented as the weekly new deaths divided by the number of habitants in a municipality per 100,000 people.

### Centro de Estudios sobre Desarrollo Economico (CEDE) database

To inform our model, we utilized municipality-level information compiled from the Observatory of Municipalities at the Center of Economic Development (CEDE) at the Universidad de Los Andes Colombia [13]. Specifically, we gathered information on municipalities’ distance from Bogotá, Colombia’s capital, in kilometers, the GDP per capita, an unsatisfied basic needs index (defined as the proportion of households that do not meet the standards of a proper household, live in overcrowding, have insufficient public services, high economic dependence, or have school-age children who do not have access to the education system); and a health policy management quality index (a score of the local governments’ management efficiency in the health sector produced by DANE, the National Statistics Department of Colombia) [14]. We calculated a density variable by taking the number of inhabitants in a municipality divided by the area of the municipality in square kilometers.

### Analysis

The above-defined databases were combined for all 76 municipalities included in the study. Using this combined panel database, four analyses were conducted: mobility trends by day of the week, mobility trends over time, a matrix of mobility trends by municipal density, and an empirical estimation of the impact of mobility on COVID-19 cases and deaths.

Mobility trends by day of the week: We used the weekly mobility trend data to understand the relation between mobility trends by days of the week. Mobility trends were divided into the four segments described above: weekends (Saturday and Sunday), shoulder days (Monday and Friday), midweek days (Tuesday, Wednesday, and Thursday) and weekly (7-day period).

Mobility trends over time: We used the weekly mobility trend data to observe patterns in average mobility trends across all 76 municipalities over time, capturing key public health and social distancing policies that were implemented over the 34.9 weeks. For context, we examined the timing of implementation of key COVID-19 policies [3].

Matrix of mobility trends by municipal density: Mobility trends were captured through a mobility time trend matrix with the following coloring categories: Municipalities that saw reduced mobility in comparison to the benchmark date of March 2 are illustrated in blue, with the darker blue showing the most reduced mobility. Municipalities that saw increased mobility are illustrated in orange, with darker orange showing more mobility.

The mobility time trend matrix was organized by time from left to right, where the left side of the matrix represented the beginning of the pandemic (March 2020) and the right side represented the end of the observation points (October 2020). As the blocks moved horizontally to the right side, each block represented one week forward to display a total of 35 blocks of which the first 34 have complete data, because information on some restrictions and exceptions were available only through October 26, 2020 (i.e., exactly 34 weeks from the start of the data). To expand upon the time dimension for reference points, we included the Start of the National Lockdown (bottom left text) and marked exception points in time according to exceptions to leave isolation according to the Colombian national government. On June 1, 2020, the government declared 43 exceptions to the National Lockdown mandate (Table 1) that included, for instance, the reopening of commerce places such as hair salons. At the bottom right of this timeline, we marked when this National Lockdown mandate ended and transitioned to the start of a “selective isolation” mandate, which initiated a period when local governments (municipalities and departments) were handed the responsibility to define their own specific exemptions for isolation measures according to their specific epidemiological situation.

**Table 1:** Timeline of exceptions to Colombia’s National Lockdown

Epidemiologic Week	Date (mm/dd/yy)	Number of Exceptions (Exc)	Principal Changes
12	3/17/20	N/A	Legal powers of the president are expanded through State of Emergency
13	3/25/20	34	Mandatory stay-at-home measures. All non-essential commerce is closed through National Lockdown
15	4/8/20	35	One additional exception to the National Lockdown
17	4/24/20	41	Six additional exceptions to the National Lockdown
19	5/6/20	N/A	Legal powers of the president are expanded
19	5/11/20	46	Outdoor physical activity is allowed in municipalities with a low number of cases
23	6/1/20	43	Reopening of commerce (e.g., hair salons)
27	7/1/20	43	Reopening of restaurants
32	8/1/20	44	One additional exception to the National Lockdown
36	8/1/20	N/A	End of National Lockdown and shift to municipality- and department-level isolation, stay-at-home, and self-care policies

Notes: See S.1 in Supplementary Material for epidemiologic week numbering. Source: [15]

### Empirical estimation

We developed an empirical model that incorporated variables from the four databases described above to estimate the relationship between mobility trends and COVID-19 cases and COVID-19 deaths. The model controlled for the following municipality-level characteristics: distance to the capital of Bogotá, GDP per capita, unsatisfied basic needs index, and health policy management quality index. The model also had a fixed effect to capture any time-invariant factors at the municipal level in Colombia as well as a monthly fixed effect:

$${\Delta }_{Yit}={\beta }_{0}+{\beta }_{1}{mob}_{it-k}+{\beta }_{2}{Oxford}_{t-k}+{\overrightarrow{\beta }}_{3}{\overrightarrow{month}}_{t}+{\overrightarrow{\beta }}_{4}{\overrightarrow{charact}}_{i}+{e}_{it}$$

In our model, *i* is the municipality, *t* is time (in weeks), *k* is the lag (1 to 3 weeks) from mobility to outcomes, Δ*y* is the change in outcomes (COVID-19 cases or deaths), *mob* is the change in mobility (in percentage points) from baseline (March 2, 2020), *Oxford* is the Oxford COVID-19 Government Response Tracker [16], *month* is a vector of characteristics associated with the month, *charact* is a vector of characteristics associated with the municipality, *β0* is a constant term to be estimated by the regression, *β1* and *β2* are scalar coefficients to be estimated through regression, *β3* and *β4* are vector coefficients to be estimated through regression, and *e* is a random error term. We estimated this model separately for the three portions of the overall mobility changes as well as the three-week segments. For each outcome and lag, the coefficient *β1* is the coefficient of primary interest, while the other coefficients control for other factors.

### Overall impacts on cases and deaths

To estimate the overall impact of various mobility restrictions on COVID-19 cases and deaths, we defined an “effective” municipality as one at the edge of the best quartile in the distribution of mobility changes for its segment of the week (i.e., the 75^th^ percentile in the reduction in mobility compared to the baseline period). For each segment of the week, we computed the reduction in mobility between a median and an effective municipality for that segment of the week. Combining these changes with the regression coefficients from our model for results which were statistically significant, we estimated the changes in resulting COVID-19 case and death rates.

Adjusting for the time periods affected by the relative portions of the week, we calculated rates per 100,000 person-years of restricted mobility. To see how the different segments of the week compared, we normalized these impacts by the overall change to get relative impacts. As these were ratios, they were not normally distributed, so we computed 95% confidence intervals using Monte Carlo simulation (with 1,000 iterations) instead of standard errors.

## Results

### Comparison among weekdays, shoulder days, and weekends

Table 2 shows the means of variables including mobility trends by day of the week segments. There are clear trends in mobility patterns according to the day of the week segment. Overall, average mobility significantly changed during weekends at -31.4% (i.e., reduction) compared to Monday and Friday mobility at -25.7% (t = -31.65, df = 2,659, *p* < 0.01). Comparing overall weekend mobility to midweek mobility at -22.2%, weekend mobility was significantly lower (t = -38.96, df = 2,659, *p* < 0.0001). This indicates that people stayed at home more often on Saturday and Sunday compared to the other day-of-week segments. Observed mobility patterns increased during the midweek, meaning people were more mobile during the midweek. The increase in mobility during the midweek is likely due to traveling for work, daily chores, and healthcare visits. Supplementary Material S.2 presents further descriptive data.

**Table 2:** Descriptive Statistics of Variables over 34.9 weeks (March 2, 2020 through Oct 31, 2020)

Weekly Segments	Observations	**Mean**	Standard Deviation	Minimum	Maximum
New Reported COVID Cases per 100,000 Inhabitants per Week	2255	**121.47**	116.01	.37	1163.17
New Reported COVID Deaths per 100,000 Inhabitants per Week	2255	**3.54**	4.15	0	49.42
Weekly Mobility	2736	**-25.70**	22.50	-76.10	226.60
Weekend Mobility	2736	**-31.40**	21.80	-81.50	162.60
Shoulder (Monday and Friday) Mobility	2660	**-24.80**	23.30	-79.60	283.00
Midweek (Tuesday—Thursday) Mobility	2660	**-22.20**	23.70	-74.30	284.60

Note*:* Mobility represents percentage point change from the starting date of March 2, 2020. All weekly segment means were significantly different from one another. All combinations were tested. See S2 in Supplementary Material for detailed methods for calculations of these variables

### Effect of National Lockdown and exceptions

**Fig. 1:**
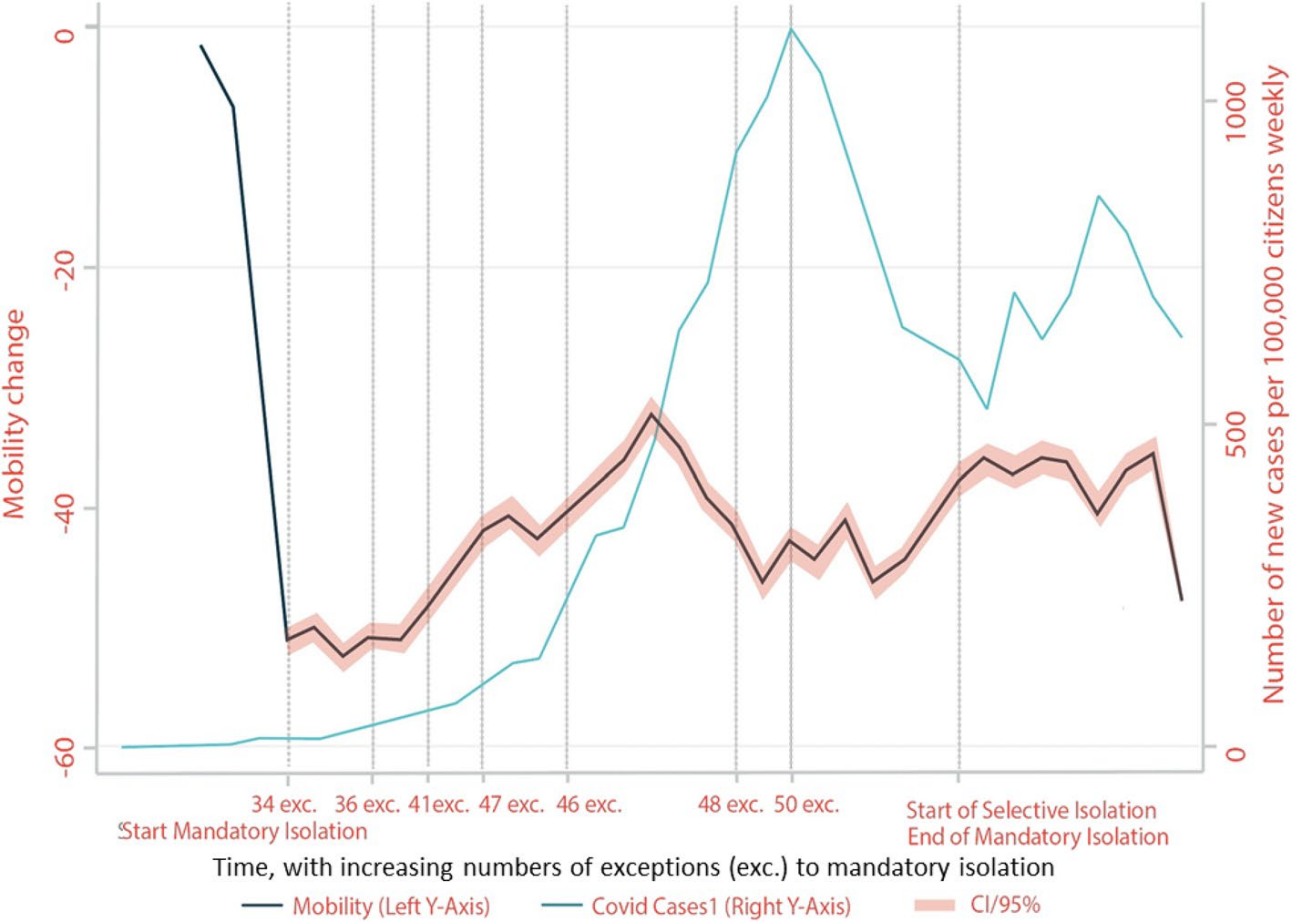
Mobility change over time compared to new COVID-19 cases nationally. Notes: The x-axis, denoting time, corresponds to the 34.9 complete weeks in the data set. The labels denote key dates in Colombia regarding exceptions to staying in mandatory isolation. Time progresses from left to right of the figure, with more exceptions declared allowing citizens to reopen businesses or travel for essential needs. The start of mandatory isolation occurred on March 25, 2020, with 34 exceptions permitting select groups to leave isolation for essential services. Other key dates included May 11, 2020, with 46 exceptions to mandatory isolation to engage in physical activity in municipalities with a low number of cases. On June 1, 2020, the government declared 43 exceptions to the stay-at-home measures that included, for example, reopening commerce places such as hair salons. On August 1, 2020, mandatory isolation for all departments in Colombia changed to selective isolation status, ending mandatory stay-at-home measures. Source: Authors’ calculations based on GRANDATA and SIVIGILA

Figure 1 shows cross-sectional trends over time in weekly mobility patterns as well as new COVID-19 cases. It shows an overall reduction in mobility when compared to the baseline of March 2, 2020. The first mandatory stay-at-home order (the National Lockdown) was declared in Colombia on March 25, 2020. As shown in Fig. 1, as time progressed after the mandatory isolation, mobility went up and down, with the number of new cases per 100,000 residents weekly steadily rising until 50 exceptions to stay-at-home measures were declared. At the end of the National Lockdown mandate, residents' mobility patterns seemed to stabilize. However, at the start of selective isolation, the number of new cases per 100,000 residents weekly rose again, though not as dramatically. While it is difficult to see a clear pattern of correlation between mobility and cases over the entire period, there does appear to be a lagged effect where cases fall a few weeks after reductions in mobility. This pattern is explored further in the empirical models presented below.

### Effect of population density

**Fig. 2:**
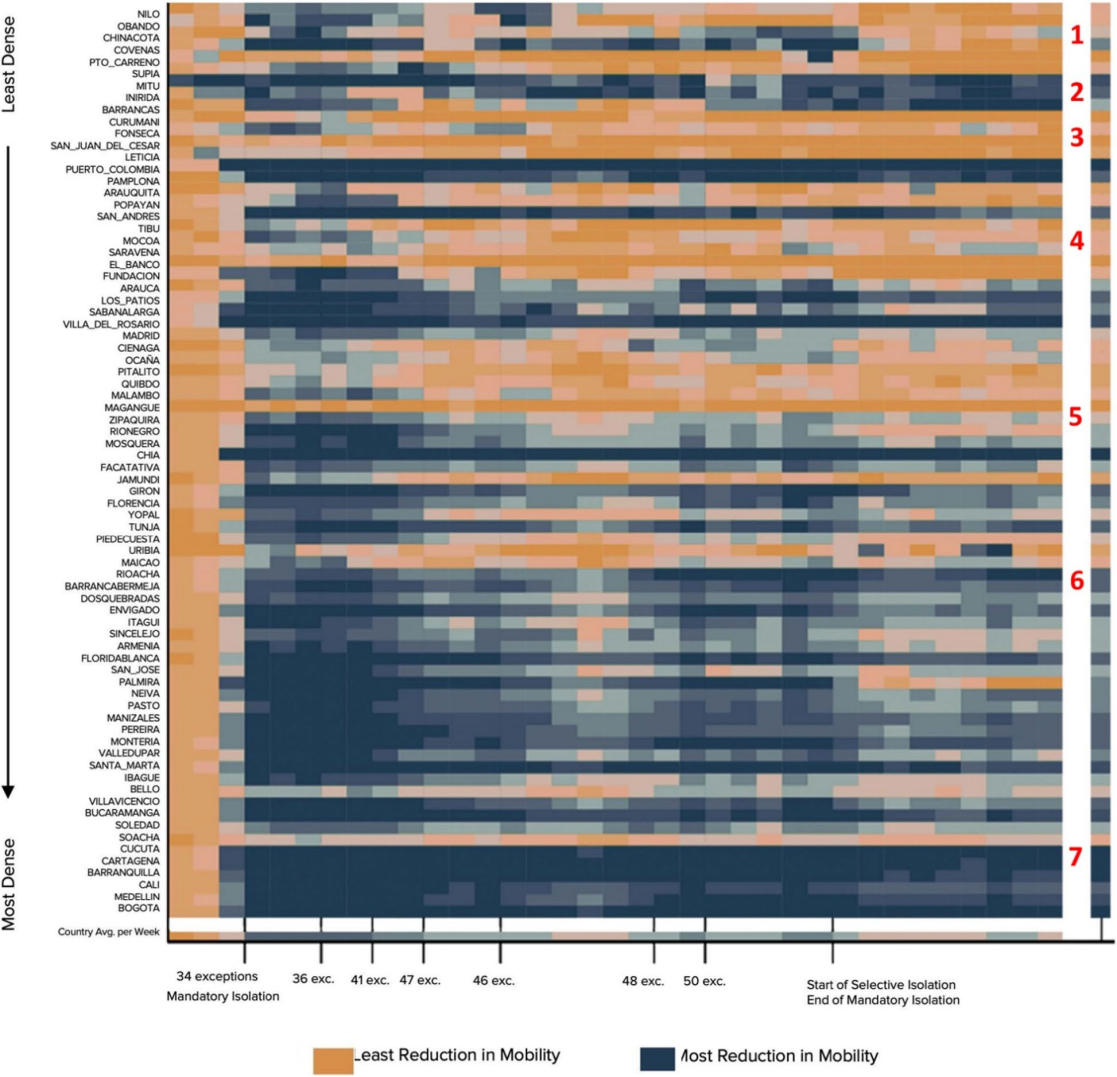
Impact of density on weekly mobility during varying COVID mobility restrictions. Notes: The right- most vertical bar (to the right of the white bar) shows the municipality’s overall mean. Source: Authors’ calculations based primarily on GRANDATA and information from DANE, the National Statistics Department of Colombia

Figure 2 shows how mobility differed by municipalities' population density and time. On the right border in Fig. 2 there is a separate vertical bar. It denotes “Municipality’s Overall Mean.” Each block represents the average overall weekly mobility of the corresponding horizontal municipality during the study. This is useful in comparing the overall averages of municipalities with each other.

On the bottom of each matrix there is a separate horizontal bar labeled “Country avg. for week.” Each block accounted for the average mobility of all the municipalities in the study for that specific week. This is useful for visualizing overall trends in the country regarding mobility in relation to government policies and announcements. The matrix is organized by the density of municipalities. The least dense municipalities are displayed at the top of the matrix and the denser municipalities are at the bottom of the matrix. We found that density and mobility were negatively correlated for all day-of-week segments (*p* < 0.01). There was a significant association between mobility municipality density [F (3, 2732) = 163.88, *p* < 0.0001]. The mean mobility reduction for the densest municipalities (mean = -35.31, SD = 14.89) was significantly different than for the least dense municipalities (mean = -14.61, SD = 20.65).

Figure 2 indicates that over time, people in highly dense cities (bottom), such as Bogotá, Medellín, Cali, and Barranquilla, displayed the most reduction in mobility (blue blocks). In general, less dense municipalities, such as Puerto Carreno, Curumani and Fonseca, displayed more variable trends in mobility and people were more mobile. The right vertical bar showing each “Municipality’s Overall Mean” illustrates the overall mean of each municipality and shows that as density increases (moving downward), people in those municipalities were less mobile (orange shifting to blue).

As shown at the bottom of Fig. 2, the country average for the week (bottom bar) demonstrates that once the National Lockdown or “Mandatory Isolation status” was implemented, mobility decreased (shift from orange to blue). Policy makers should view blue blocks as municipalities that were adhering better to stay-at-home measures and reducing their mobility patterns (staying at home) more. Orange blocks indicate that people were not following stay-at-home guidelines and were continuing mobility over time. For municipalities that have consistent orange blocks observed over the study, these trends show that people in those areas remained mobile throughout the pandemic.

The numbers 1 through 7 in the white column on right relate to the following comments about successive groups of municipalities of increasing density**.** (1) The 5 municipalities at the top of the figure (Nilo through Puerto Carreno) have the lowest population density. The preponderance of orange lines indicate that residents reduced their mobility relatively little. Because of the low population density, workplaces, markets, shopping, and residential areas tend to be far apart so residents needed to travel extensively for normal activities and had little scope to reduce their mobility. (2) The next 3 municipalities (Supia through Inírida) have mostly blue bars. This means that their population’s mobility declined substantially from the start of the pandemic, despite their low population density. Possible explanations include: more stringent restrictions on mobility by the municipal government, adverse impacts on employment and income, or greater adherence by residents. (3) The next 14 municipalities (Barracas through El Banco) have mostly orange bars (corresponding to high mobility). One exception, however (Leticia), is completely blue after the second week, likely due to data gaps during the follow up period. Gaps in mobility data get processed as zero mobility, corresponding to a maximum reduction in mobility. (4) The next 10 municipalities (Fundacion through Marangue) represent lower-middle density. Still displaying a predominantly orange pattern (lowest reduction in morbidity), most of these municipalities generally exhibit substantial reductions in mobility. (5) The following 14 cities (Zipaquira through Maicao) represent middle density. Depicted with a range of colors, including a substantial amount of light blue-gray, they represent variable, but generally modest, changes in mobility. (6) The next 22 municipalities (Rioacha through Soacha) represent upper middle density. Depicted with substantial medium blue, they show moderate to high reductions in mobility. As density increases, workplaces, markets, shopping and residential areas all become closer, allowing residents to curtail their mobility to moderate levels. (7) The 6 most dense cities (Cucuta through Bogota) all appear in varying shades of dark blue, indicating that all had substantial reductions in mobility throughout the pandemic (the opposite pattern from the least dense cities). In these dense cities, workplaces, markets, shopping and residential areas are likely within a few kilometers of one another. Therefore, residents were able to substantially reduce their mobility while still meeting daily needs. Notes: See S.3 in Supplementary Material for more information on the coloring of Fig. 2 and details regarding outliers.

For policy makers, these results demonstrate that in Colombia implementing a national lockdown strongly discouraged movement and resulted in reduced mobility (blue blocks). As time progressed and more exceptions were made, the country’s overall average mobility remained negative. Mobility did increase for the country overall between the announcements of 46 and 48 exceptions but returned to reduced observations after the 48 exceptions were announced. Once the end of mandatory isolation and the start of selective isolation were announced, mobility increased (shifted to orange) in lower-density municipalities.

### COVID-19 Cases

As weekly and weekend mobility increased, Table 3 shows that the future positive number of new COVID-19 cases increased significantly two weeks later. For example, if weekly mobility patterns increase by one percentage point, the weekly number of new cases increased by 0.16 cases per 100,000 people two weeks later (*p* < 0.10). This increase was more dramatic for weekend mobility. If mobility patterns on the weekend increase by one percentage point, the weekly number of new cases per 100,000 people two weeks later increases by 0.31 cases per 100,000 people (*p* < 0.01). When alternative lags (one and three weeks) were examined, the results for subsequent cases were no longer statistically significant for both weekly and weekend mobility segments.

**Table 3:** Day of week mobility segments correlates to future COVID-19 cases

	**(1) Cases one week later**	**(2) Cases two weeks later**	**(3) Cases three weeks later**
**7 Day Weekly Mobility**	0.089	0.160*	-0.015
Standard Errors	(0.090)	(0.097)	(0.096)
Observations	1,582	1,641	1,699
R^2^	0.027	0.026	0.044
**Weekend Mobility**	0.124	0.305***	0.047
Standard Errors	(0.116)	(0.112)	(0.111)
Observations	1,582	1,641	1,699
R^2^	0.027	0.029	0.044
**Shoulder Day Mobility**	0.065	0.050	0.072
Standard Errors	(0.092)	(0.090)	(0.088)
Observations	1521	1580	1633
R^2^	0.026	0.023	0.032
**Midweek Mobility**	0.024	0.085	0.003
Standard Errors	(0.090)	(0.088)	(0.087)
Observations	1521	1580	1633
R^2^	0.026	0.023	0.031

Note: Standard errors in parentheses. Includes departmental fixed effects. Cases presented in the number of new cases per 100,000 people. ***p < 0.01, **p < 0.05, *p < *0.10.* Source: authors calculations

### COVID-19 Deaths

Mobility was correlated with future deaths from COVID-19. As weekly mobility increased overall, the future positive number of new deaths from COVID-19 cases increased two weeks later (see Table 4). At a larger magnitude, as weekend mobility increased, the future number of deaths increased significantly at one and two weeks later. For example, if weekly mobility patterns increased by 10 percentage points, the weekly number of new deaths per 100,000 people two weeks later increased by 0.11 deaths per 100,000 people. This increase was more dramatic for weekend mobility. If mobility patterns on the weekend increased by 10 percentage points, the weekly number of new deaths per 100,000 people one week later increased by 0.13 deaths per 100,000 people. At two weeks later, the weekly new deaths per 100,000 people increased by 0.16 deaths per 100,000 people. The results for deaths one week later and three weeks later were not significant for weekly mobility. The results for deaths three weeks later for weekend mobility also were not significant.

**Table 4:** Day of week mobility segments correlates to future deaths from COVID-19

	**(1) Deaths one week later**	**(2) Deaths two weeks later**	**(3) Deaths three weeks later**
**7-Day Weekly Mobility**	0.0075	0.0112**	0.0011
Standard Errors	(0.0048)	(0.0047)	(0.0047)
Observations	1,582	1,641	1,699
R^2^	0.0220	0.0240	0.0200
**Weekend Mobility**	0.0132**	0.0165***	0.0038
Standard Errors	(0.0056)	(0.0055)	(0.0054)
Observations	1,582	1,641	1,699
R^2^	0.024	0.026	0.021
**Shoulder Day Mobility**	0.0059	0.0066	0.0035
Standard Errors	(0.0044)	(0.0043)	(0.0043)
Observations	1521	1580	1633
R^2^	0.0230	0.0210	0.0210
**Midweek Mobility**	0.0043	0.0052	0.0032
Standard Errors	(0.0043)	(0.0043)	(0.0042)
Observations	1521	1580	1633
R^2^	0.0230	0.0210	0.0210

Note: Standard errors in parentheses. Includes departmental fixed effects. Deaths presented in the weekly number of new deaths per 100,000 people. ***p < 0.01, **p < 0.05, *p < 0.1. Source: Authors’ calculations

Two segments of the week showed statistically significant impacts of mobility on COVID-19 cases and deaths—overall mobility and weekend mobility. Table 5 derives the resulting impact. We found that the impact per 100,000 person years of weekend mobility was substantially and significantly greater than for overall mobility. Specifically, the relative impact per 100,000 person years of weekend mobility (and 95% confidence intervals) compared to all days are 6.40 (1.99–9.97) for cases and 4.94 (1.33–19.72) for deaths (see S.4 and S.5 in Supplementary Material for further details on calculations).

**Table 5:** Derivation of impact of mobility restriction on COVID-19 cases and deaths

Line	Item	All days		Weekend days	
		Estimate	Standard error of the estimate	Estimate	Standard error of the estimate
(1)	Mobility: Percentage point reduction from March 2, 2020				
(2)	Best quartile municipality	40.60	n.a	46.65	n.a
(3)	Median municipality	28.24	n.a	34.80	n.a
(4)	Difference (potential improvement) [(2)-(3)]	12.36	n.a	11.85	n.a
(5)	Regression coefficient of mobility on municipality's weekly rate per 100,000 population of:				
(6)	Reported COVID-19 cases	0.1600	0.0097	0.3050	0.1120
(7)	Reported COVID-19 deaths	0.0112	0.0047	0.0165	0.0055
(8)	Impact of potential improvement in mobility on municipality's weekly rate per 100,000 population on:				
(9)	COVID-19 cases [(4) x (6)]	1.9776	0.1199	3.6143	1.3272
(10)	COVID-19 deaths [(4) x (7)]	0.1384	0.0581	0.1955	0.0652
(11)	Days per week restricted	7.00	0.00	2.00	0.00
(12)	Weeks per year	52.00	0.00	52.00	0.00
(13)	Potential improvement in municipality's rate per 100,000 person-years of restricted mobility on:				
(14)	COVID-19 cases [(9) x (12) / (11)]	14.69	0.89	93.97	34.51
(15)	COVID-19 deaths [(10) x (12) / (11)]	1.03	0.43	5.08	1.69
(16)	Relative potential improvement in municipality's rate per 100,000 person-years of restricted mobility on:^a^				
(17)	COVID-19 cases [derived from (14)]	1.00	n.a	6.40	n.a
(18)	COVID-19 deaths [derived from (15)]	1.00	n.a	4.94	n.a

^a^Central estimates (and 95% confidence intervals) for relative potential impact for weekend person years compared to all days are 6.40 (1.99–9.97) for cases and 4.94 (1.33–19.72) for deaths

### Simulation of impact of mobility restrictions

Table 6 estimates the impact on national aggregate COVID-19 cases and deaths if mobility had been reduced to the level in effective municipalities throughout Colombia through October 31, 2020, using the “all days” impact. It shows that 17,145 cases and 1,209 deaths would have been averted. While the shares of these total events are modest (1.63% and 3.91%, respectively), the estimated reductions are statistically significant.

**Table 6:** Estimated impact of reduced mobility in Colombia

Line	Description	Value	Standard error	Source
(1)	Impact of potential reduction in mobility on municipality's weekly rate per 100,000 population			
(2)	Reported COVID-19 cases	1.9776	0.1199	Table 5
(3)	Reported COVID-19 deaths	0.1384	0.0581	Table 5
(4)	Average municipality weekly rate per 100,000 population (3/1/20 through 10/31/20)			
(5)	Reported COVID-19 cases	121.47	n.a	Table 4
(6)	Reported COVID-19 deaths	3.54	n.a	Table 5
(7)	As percentage of average weekly rate			
(8)	Reported COVID-19 cases	1.63%	0.10%	(5) / (8)
(9)	Reported COVID-19 deaths	3.91%	1.64%	(6) / (9)
(10)	Aggregate cumulative numbers through 10/31/20			
(11)	Reported COVID-19 cases	1,053,122	n.a	WHO (2022)
(12)	Reported COVID-19 deaths	30,926	n.a	WHO (2022)
(13)	Projected national COVID-19 cases and deaths that would have been averted through 10/31/20 through greater mobility reductions			
(14)	Number of COVID-19 cases averted	17,145	1,039	(8) x (11)
(15)	Number of COVID-19 deaths averted	1,209	508	(9) x (12)

Source: WHO (2022) [1]

## Discussion

### Main contributions

This study used mobility data to capture key changes in actual patterns of mobility over 34.9 weeks during the COVID-19 pandemic in Colombia and used these changes in mobility patterns to understand the impact on COVID-19 cases and deaths. A strength of the study is its focus on changes in per-person mobility rather than the absolute level. The available GRANDATA lack detailed data about the myriad characteristics of the municipalities on dimensions such as population density, demographics, transportation systems, mobile phone penetration, etc. Nevertheless, the study's design, which is based on reductions in mobility, controls for all municipality-level characteristics. Over the study duration of under one year, these factors likely changed little, so that reductions in mobility are related primarily to pandemic-related responses.

The study is unique in that the mobility data were analyzed by different day-of-the-week segments to understand how mobility changed throughout parts of the 7-day week. The results of analysis show that reductions in both weekend and weekly mobility were correlated to reductions in future cases and deaths. However, weekend mobility reductions (measured in 100,000 person years) had 6.40 times and 4.94 times larger impact on cases and deaths, respectively, than all days of the week combined. These results provide useful evidence that policy makers can use to curb cases and deaths from COVID-19 or a future pandemic, while trying to lessen the toll mobility restrictions have on the economy.

### Comparisons to previous studies

Our statistical findings report that people were less mobile in municipalities that are more densely populated, such as Bogotá, Medellín, Cali, and Barranquilla (See Fig. 2). In municipalities that are less densely populated, such as Puerto Carreno, Curumani, and Fonseca, observed mobility went up over time. These results are consistent with a study on human mobility trends in the United States during COVID-19 that reported more dense states reduced their mobility more noticeably, while less dense states displayed increased mobility patterns [17]. Reduced mobility in highly-populated municipalities could be attributed to people traveling less in order to go to work or for essential needs. Additionally, because there was a greater probability that someone in a dense municipality would contact people outside or during everyday tasks, people in denser municipalities could have reduced mobility more to adhere to social distancing measures.

In Colombia the distribution of population density across municipalities was highly correlated with important structural characteristics. Thus, observed higher mobility reductions in more dense municipalities could be explained by additional factors (other than work-home geographical proximity). These include a larger prevalence of economic activities in which work-from-home is feasible, a greater proportion of middle- and higher-income households that have enough economic protection to adhere to stay-at-home measures, a more developed network of delivery services, and better internet connectivity that made it possible to work from home and conduct a virtual life.

Our findings also report that people on average were less mobile during the weekend compared to weekday segments in the observed municipalities (see Table 1). Although our study does not account for the reason people were staying in or going out more, the findings are consistent with the results in a study on risk attitudes and mobility during the COVID-19 pandemic in 58 countries. That study found that, on average, during the weekends there was a greater reduction in visits to retail and recreational places, grocery and pharmacy, parks, and transit stations, compared to weekdays. Examining how the density of an area impacts mobility, the study found that for countries with a higher population density, there was a significant decline in visits to grocery and pharmacy, transit stations, and workplaces [18]. While our municipality-level study did not track the types of locations that people go to, the findings on reduced visits for essential needs, travel, and work in dense locations aligned with conclusions that mobility patterns in highly dense municipalities were reduced the most compared to less dense municipalities (see S.6 in Supplementary Material for further results).

The impact on COVID cases and deaths from reductions in mobility has been analyzed in other studies, showing large impacts on COVID-19 infection rates in comparison to the absence of anti-contagion policies [19] and variation in non-pharmaceutical interventions across Europe [20]. While these studies are extremely important, they estimate the impact of actual mobility through varying government policies that limit mobility. The results presented in this study show an impact on COVID-19 cases and deaths using variation in observed mobility trends. Analyzing observed mobility, we were also able to understand the impact of different weekly segments, showing that reducing weekend mobility was more important than reducing mobility over all seven days of the week.

The larger correlation of weekend mobility and future cases and deaths in our model can be attributed to the idea that if people were mobile on the weekends, they could have been in less formal settings where proper social distancing and safety measures were not as strictly enforced or it was not possible to keep a mask on all the time (e.g., visits with family or friends, bars, and restaurants). This mobility led to increased risk of exposure to COVID-19. Our discoveries are consistent with a study observing the 7-day cycle in COVID-19 infection and mortality rates. That study reported that people may become infected at higher rates from weekend activity because of increased social interactions during the weekend [21]. As a result, they found that vulnerable populations exhibit signs of COVID-19 infection at higher rates five days after the weekend. This result aligns with our findings that increased weekend mobility led to increased future cases and deaths. In comparison, during the week, although people were more mobile, they could have been in scenarios where strict health protocols were enforced (i.e., workplaces, doctor offices, essential businesses). Our study is novel in that it combines multiple databases to create a model that predicts future cases and deaths by mobility patterns during the weekend or weekday.

### Limitations

There are several limitations to our study. The mobility data provided for our analyses does not distinguish between Venezuelans and Colombians specifically, making it difficult to determine mobility patterns by nationality. The data do not include demographic information such as age, gender, and occupation of the users observed. Although our analysis by day-of-the-week did not adjust for holidays, this effect was probably small. During the complete study period, only 1 of 102 midweek days (i.e., 1.0%) and 11 of 68 (i.e., 16.2%) shoulder days were public holidays. However, about half of Colombians work in the informal sector and may not strictly observe public holidays, so holidays during shoulder days likely had limited impact. Further, the mobility data did not identify the purpose of individuals leaving their house and their final destination during the observed movement. This limited the ability to analyze mobility patterns by visits for different purposes, such as work, school, health care, exercise, or visiting friends or family. Some mobility data, which link with known sites such as parks, stadiums, or workplaces, could offer such additional granularity [22].

The rapidly changing COVID-19 pandemic made it hard to evaluate the National Lockdown and the increasing exceptions. In trying to interpret the rises and falls in rates of COVID-19 cases, the onset of new virus variants and varying number of susceptible persons were confounded with policy modifications. As many lockdown and exceptions policies were national, the availability of municipal-level data for 76 cities did not add as much statistical power as the number of observations might have suggested.

Although we hypothesized that mobility affected subsequent COVID-19 cases and deaths, reverse causality (COVID-19 perceptions affect mobility) is possible. We controlled for this possibility by examining each municipality's COVID-19 outcome rates one, two and three weeks later than its observed mobility, but this approach may have been insufficient. Nevertheless, the mobility data used in this model still provide substantial information to examine mobility in relation to time, cases, and deaths.

### Future research

Two further questions especially deserve future research. First, how do findings vary according to characteristics of the municipality? Weekend mobility might be most important in denser or wealthier municipalities, where opportunities for social gatherings might be more plentiful. Midweek mobility might be more important in municipalities where the informal sector is relatively larger and where a smaller share of workers could work remotely. Findings from such ecological analyses could shape policies for this or possible future variants or pandemics to be customized to the municipalities' characteristics.

Second, how should these findings be adapted to the current situation where vaccines have become widely available? Understanding and reporting on these trends is imperative for controlling the current pandemic and informing policies in potential future pandemics. New variants and the decay of immunity may erode vaccine protection. Even if legal restrictions on mobility were relaxed, operators of social venues and individual households may wish to use the findings to protect themselves, their friends, and clients. Thus, understanding mobility-based policies examined in this paper to reduce exposure remain highly relevant for controlling the current pandemic and informing policies in potential future pandemics.

## Conclusion

This study found that greater reductions in mobility led to significantly fewer COVID-19 cases and deaths in Colombia two weeks after restrictions were announced. If all municipalities at the median had reduced mobility to the level in “effective” municipalities, we estimate that Colombia would have averted 17,145 cases and 1,209 deaths over the 34.9-week period. These numbers, while impressive, represent only modest shares of COVID-19 cases (1.63%) and deaths (3.91%). The study found strikingly large impacts of reductions in weekend mobility compared to overall mobility. On the basis of person years, restrictions on weekend activities were 6.40 times as effective on cases and 4.94 times as effective on deaths as those on all days. We also expect that weekend restrictions are less disruptive to the economy, as they would allow factories and many other in-person business to continue to operate. Thus, government leaders may wish to create and strongly enforce policies to restrict weekend mobility, with less emphasis on policies limiting weekday polices.

## Supplementary Information

**Additional file 1. **

## Data Availability

Most of the data that support the findings in this study are available from publicly available sources, as follows: (1) Population data are available from: Department Administrative Nacional Estastica (DANE). Colombia. Population by Municipality and Age. Annex on population projections by municipality with simple ages, 2018–23 (Anexos-proyecciones-poblacion-municipios-edadessimples-2018–2023). [cited 2022 Jan 1]. Available from: https://www.dane.gov.co/index.php/estadisticas-por-tema/demografia-y-poblacion/proyecciones-de-poblacion. (2) Numbers of COVID-19 cases and deaths come from SIVIGLA (Sistema Nacional de Vigilancia en Salud Pública). Instituto Nacional de Salud. 2021, Available from: https://www.ins.gov.co/Direcciones/Vigilancia/Paginas/SIVIGILA.aspx. (3) Dates and details about government lockdowns and exceptions come from: Los Andes School of Government. Policy Response Database. 2021, Available from: https://github.com/dccontreras/Colombia-covid-policy. The mobility data by municipality and day come from GRANDATA, but restrictions apply to the availability of these data, which were used under license for the current study, and so are not publicly available. Inquiries regarding access may be directed to: United Nations Development Programme [UNDP]. Grandata Initiative. 2020, Available from: https://www.undp.org/latin-america/grandata.

## Abbreviations

CDC: Centers for Disease Control and Prevention
CEDE: Centro de Estudios Sobre Desarrollo Económico (Center for Studies on Economic Development, University of the Andes)
DANE: Departamento Administrativo Nacional Encargado (National Statistics Department of Colombia)
ELRHA: Enhancing Learning and Research for Humanitarian Assistance
INS: Instituto Nacional de Salud (National Institute of Health)
GRANDATA: Mobility data from UNDP
MADID: Mobile Advertising ID
SIVIGILA: Sistema Nacional de Vigilancia en Salud Pública (National Epidemiologic Surveillance System)
UNDP: United Nations Development Program
WHO: World Health Organization
