# Supplementary material for: Impact of weekday and weekend mobility and public policies on COVID-19 incidence and deaths across 76 large municipalities in Colombia: statistical analysis and simulation
Source: BMC Public Health. 2022 Dec 31;22:2460. doi: 10.1186/s12889-022-14781-7 (PMC9803892; doi:10.1186/s12889-022-14781-7)
Supplement: Supplementary file 1 — Additional file 1. [file 12889_2022_14781_MOESM1_ESM.docx]

# **Supplementary Material**

**for**

**Impact of Weekday and Weekend Mobility and Public Policies on COVID-19 Incidence and Deaths across 76 Large Municipalities in Colombia: Statistical Analysis and Simulation**

Jamie S. Jason^1^

Diana M. Bowser^1^

Arturo Harker Roa^2^

Diana C. Contreras Ceballos^2^

Santiago Muñoz^2^

Anna G. Sombrio^1^

Donald S. Shepard^1^,*

1 The Heller School for Social Policy and Management, Brandeis University. United States.

2 School of Government Alberto Lleras Camargo, Universidad de Los Andes. Colombia.

*Corresponding author: shepard@brandeis.edu

# **Supplementary Material**


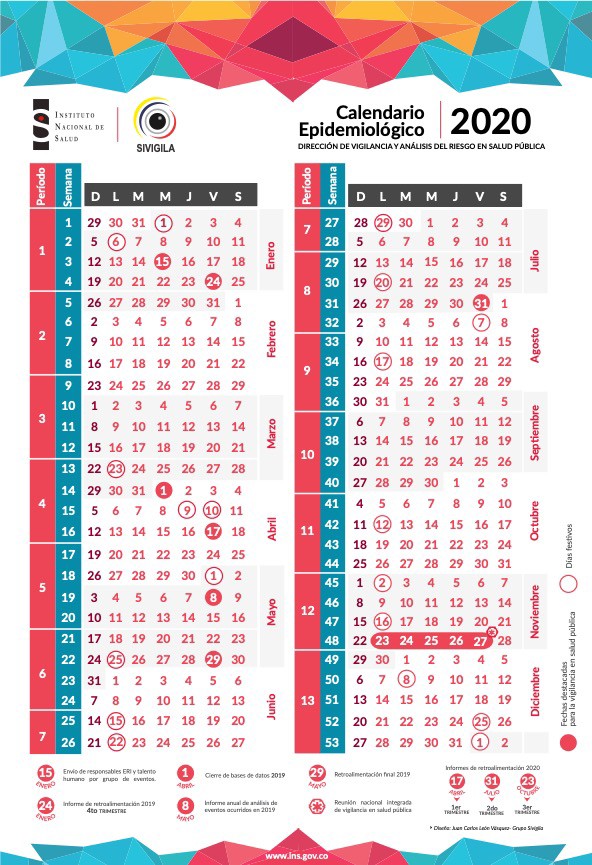
**S.1 Week Numbering Guide**

Source: SIVIGILA, 2021 [1]

**S.2 Summary Statistics on Density and Methodological Notes**

Notes: 7-Day Weekly Mobility was calculated by averaging the observed mobility averages by all weeks together for all municipalities in Colombia. The mean of -25.66 is a percentage showing that in all of Colombia throughout the study, weekly mobility on average reduced by 25.66% from the benchmark metric on March 2, 2020.

Weekend mobility was calculated by averaging the observed mobility on Saturday and Sunday by all weeks together for all municipalities in Colombia. The mean of -31.38 is a percentage showing that in all of Colombia throughout the study, weekend mobility on average reduced by 31.38% from the benchmark metric on March 2, 2020. Comparing average percentage changes by the day of week segments, mobility patterns were reduced the most on weekends, indicating that people stayed at home more during the weekends.

Shoulder day mobility was calculated by averaging the observed mobility on Monday and Friday by all weeks together for all municipalities in Colombia. The mean of -24.79 is a percentage showing that in all of Colombia throughout the study, shoulder day mobility on average reduced by 24.79% from the benchmark metric on March 2, 2020.

Midweek mobility was calculated by averaging the observed mobility on Tuesday, Wednesday, and Friday by all weeks together for all municipalities in Colombia. The mean of -22.18 is a percentage showing that in all of Colombia throughout the study, midweek mobility on average fell by 22.18% from the benchmark metric on March 2, 2020.

The density variable was calculated by taking the number of inhabitants in a municipality according to SIVIGILA [1] divided by the squared kilometer area of the municipality according to CEDE. Thus, the average density of municipalities in Colombia was 1,304.23 habitants per square kilometer.

The new weekly cases variable was calculated using weekly numbers from the National Institute of Health (INS) in Colombia. The average number of new weekly cases in all of the 76 municipalities studied was 682.29 cases per week.

Population figures were used to calculate new weekly COVID-19 cases and deaths rates per 100,000 population of the 76 municipalities studied. The new cases variable was presented as the weekly new cases of COVID-19 according to the new weekly cases from the INS divided by the number of inhabitants in a municipality per 100,000 people. The mean of new cases per 100,000 people is 121.47 averaging the mean of all weeks together for all municipalities. If a municipalities’ new cases per 100,000 average for all weeks combined were less than 121.47, their new cases per 100,000 number would fall below average indicating that municipality is performing better than the average of the country in stopping the spread of COVID-19.

The mean of new deaths per 100,000 people is 3.54 averaging the mean of all weeks together for all municipalities. The new deaths variable was presented as the weekly new deaths divided by the number of inhabitants in a municipality per 100,000 people. If a municipalities’ death toll per 100,000 average for all weeks combined were less than 3.54, their new deaths per 100,000 number would fall below average indicating that municipality is performing better than the average of the country in slowing the number of deaths from COVID-19. Source: Authors’ calculations.

**S.3 Note on Figure 2**

The coloring of the blocks was scaled according to the minimum mobility in the study. Municipalities that reduced mobility are illustrated in blue with the darker blue showing the most reduced mobility. Municipalities that increased mobility are illustrated in orange, with darker orange showing more mobility.

***Municipalities displaying outliers in Figure 2***

The following municipalities displayed different mobility data than municipalities with similar density characteristics. These outliers reduced mobility overall (blue) instead of displaying the pattern of increased mobility (orange): Covenas, Mitu, Inirida, Barrancas, Puerto Colombia, and Pamplona. For policy makers, these municipalities adhered to stay-at-home measures better.

These outliers increased mobility overall (orange) instead of displaying the pattern of reduced mobility (blue): Jamundi, Yopal, Tunja, Piedecuesta, Bello, and Soacha. For policy makers, people in these municipalities did not adhere to stay-at-home measures and were more mobile in general.

**S.4 Calculations for New Cases and Deaths Avoided by Reducing Mobility**

We calculated how a municipality moving from the median mobility average to the lower quartile mobility average will impact the number of new cases and deaths weekly according to the coefficients in our model.

For example, to calculate the 7-day weekly mobility numbers we first found the percent change from the median mobility to the lower quartile. The median and lower quartile values were 28.24 and 40.60 percentage point reductions from baseline. Their difference is 12.36 percentage points. Then we multiplied the percent change by the coefficient (.160) in the model two weeks later for 7-day weekly mobility getting 1.98 new weekly cases per 100,000 population two weeks later. This reduction in cases corresponds to 1.63% of the weekly average (121.47) and an effect size of 1.71% (as a share of the standard deviation of weekly cases of 21.80).

**S.5 Average Daily Rates of New Cases and Deaths per 100,000 People**

|  | Mean | Std. Dev. | Lower Quartile | Median | Upper Quartile | Min | Max |
| --- | --- | --- | --- | --- | --- | --- | --- |
| New Cases per Day | 17.92 | 17.34 | 5.3 | 13.76 | 24.91 | .03 | 166.17 |
| New Cases per Weekend | 53.76 | 52.01 | 15.89 | 41.29 | 74.73 | .09 | 498.5 |
| New Cases per Weekday | 71.68 | 69.35 | 21.18 | 55.05 | 99.64 | .12 | 664.67 |
| New Deaths per Day | .49 | .58 | .08 | .32 | .67 | 0 | 7.06 |
| New Deaths per Weekend | 1.47 | 1.75 | .25 | .97 | 2 | 0 | 21.18 |
| New Deaths per Weekday | 1.96 | 2.33 | .34 | 1.29 | 2.66 | 0 | 28.24 |

Notes: The New Cases per Day variable per 100,000 population was calculated by taking the cases variable (The number of new weekly cases from the National Institute of Health (INS) divided by the number of habitants in each municipality) and dividing the weekly cases by 7. To find the new cases per weekend day per 100,000 population, the new cases per day number was multiplied by 4. To calculate the new cases per weekday per 100,000 population, the new cases per day number was multiplied by 3. The same process was applied to calculate new deaths per day, weekend day, and weekday.

Source: Authors’ calculations

**S.6 Relationship of Mobility to Travel Index**

1. **Average actual mobility by reported travel for work**


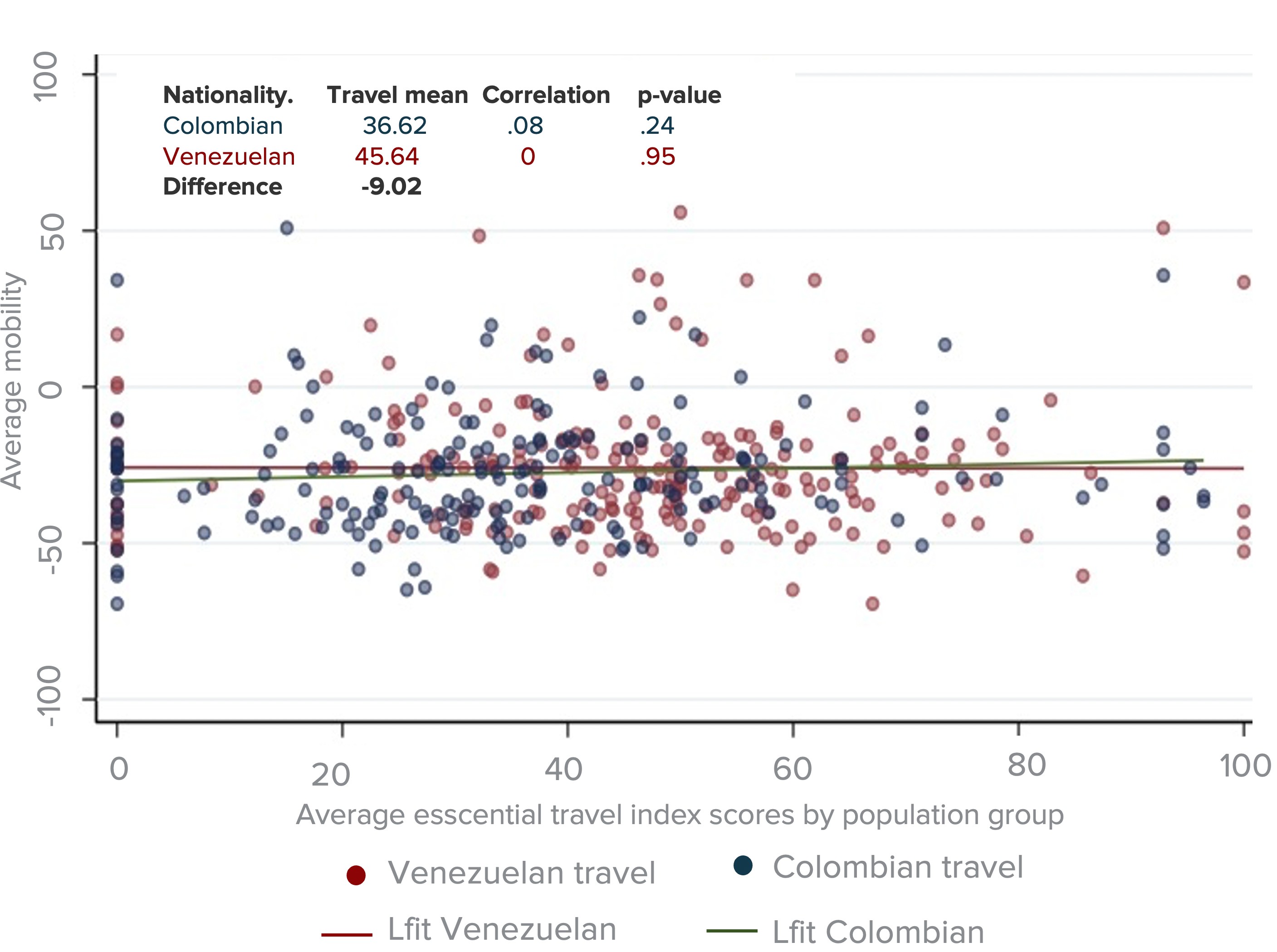


1. **Average actual mobility by reported essential travel**


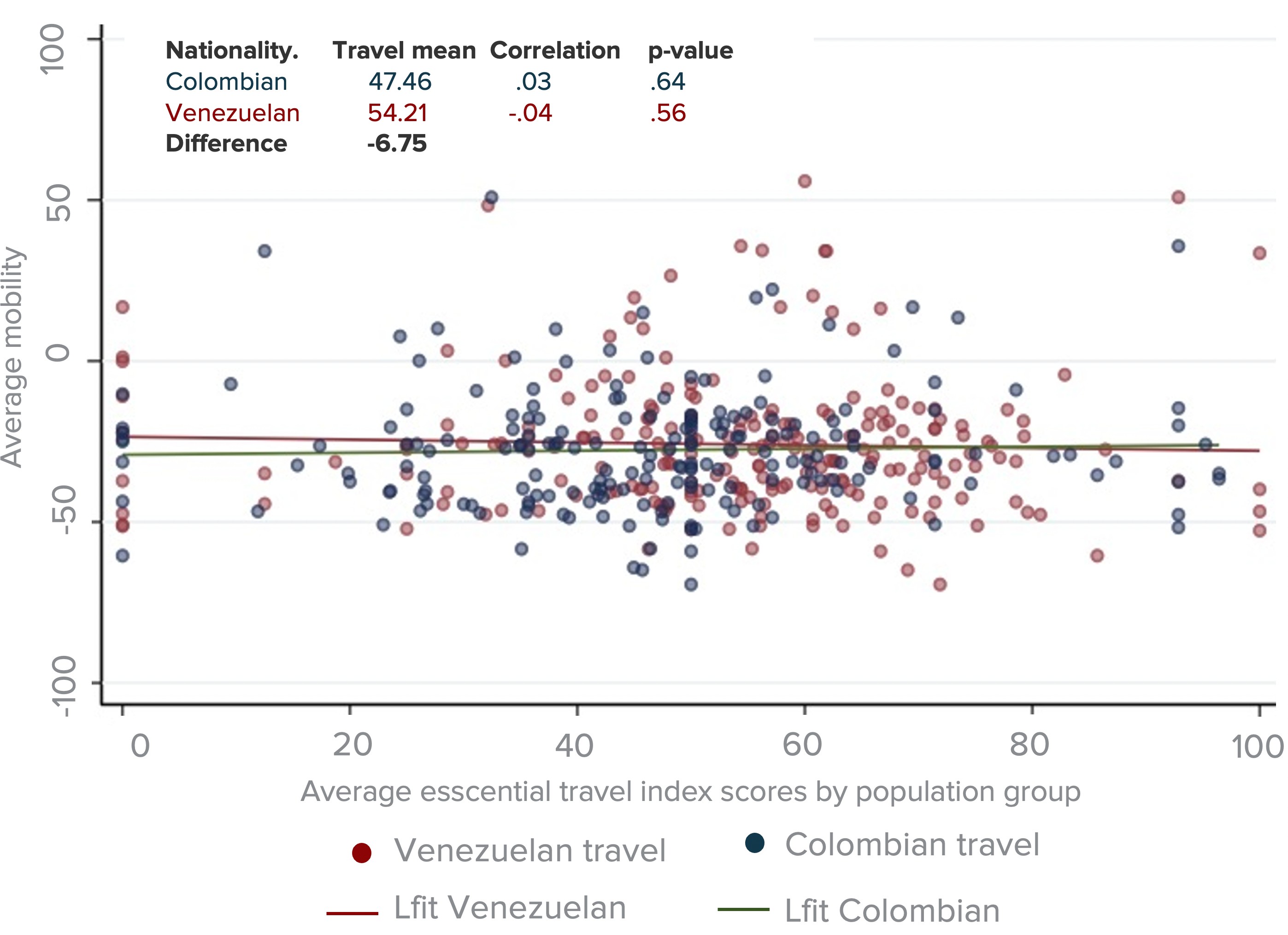


Source: Authors’ calculations

**REFERENCES**

1. SIVIGLA (Sistema Nacional de Vigilancia en Salud Pública). Instituto Nacional de Salud. 2021, Available from: <https://www.ins.gov.co/Direcciones/Vigilancia/Paginas/SIVIGILA.aspx>.
